# Nationwide retrospective study of critically ill adults with sickle cell disease in France

**DOI:** 10.1038/s41598-021-02437-2

**Published:** 2021-11-30

**Authors:** Maïté Agbakou, Armand Mekontso-Dessap, Morgane Pere, Guillaume Voiriot, Muriel Picard, Jérémy Bourenne, Stephan Ehrmann, Emmanuel Canet, Alexandre Boyer, Saad Nseir, Fabienne Tamion, Arnaud W. Thille, Laurent Argaud, Emmanuel Pontis, Jean-Pierre Quenot, Francis Schneider, Arnaud Hot, Gilles Capellier, Cécile Aubron, Keyvan Razazi, Agathe Masseau, Noëlle Brule, Jean Reignier, Jean-Baptiste Lascarrou

**Affiliations:** 1 Service de Médecine Intensive Réanimation, Centre Hospitalier Universitaire Hôtel-Dieu, 30 Bd. Jean Monnet, 44093 Nantes Cedex 1, France; 2 Médecine Intensive Réanimation, Hôpital Henri Mondor, Assistance Publique des Hôpitaux de Paris, Créteil, France; 3 Plateforme de methodologie et biostatistique, Direction de la Recherche de l’Innovation, Centre Hospitalier Universitaire de Nantes, Nantes, France; 4 Sorbonne Université, Assistance Publique Hôpitaux de Paris, Service de Médecine Intensive et Réanimation, Hôpital Tenon, Paris, France; 5 Réanimation Polyvalente, Institut Universitaire du Cancer de Toulouse-Oncopole, CHU Toulouse, Toulouse, France; 6 Médecine Intensive Réanimation, Réanimation des Urgences, CHU la Timone 2, Marseille, France; 7 Médecine Intensive Réanimation, INSERM CIC 1415, CRICS-TriggerSEP Research Network, Centre Hospitalier Régional Universitaire de Tours and Centre d’Etude Des Pathologies Respiratoires (CEPR) INSERM U1100, Université de Tours, Tours, France; 8 Médecine Intensive Réanimation, Hôpital Pellegrin, Centre Hospitalier Universitaire de Bordeaux, Bordeaux, France; 9 Médecine Intensive-Réanimation, CHU de Lille; Inserm U1285, Univ. Lille, CNRS, UMR 8576-UGSF-Unité de Glycobiologie Structurale et Fonctionnelle, 59000 Lille, France; 10 Médecine Intensive Réanimation, Hôpital Charles Nicolle, Centre Hospitalier Universitaire de Rouen, Rouen, France; 11 Médecine Intensive Réanimation, Centre Hospitalier Universitaire de Poitiers, Poitiers, France; 12 Médecine Intensive Réanimation, Hôpital Edouard Herriot, Hospices Civils de Lyon, Lyon, France; 13 Réanimation Médicale, Centre Hospitalier Universitaire de Rennes, Rennes, France; 14 Médecine Intensive Réanimation, Centre Hospitalier Universitaire de Dijon, Dijon, France; 15 Médecine Intensive Réanimation, Hôpital de Hautepierre, Centre Hospitalier Universitaire de Strasbourg, Strasbourg, France; 16 Médecine Intensive Réanimation, Hôpital de la Croix Rousse, Hospices Civils de Lyon, Lyon, France; 17 Médecine Intensive Réanimation, Centre Hospitalier Régional Universitaire de Besançon, Besançon, France; 18 Réanimation Médicale, Hôpital de la Cavale Blanche, Centre Hospitalier Régional Universitaire de Bretagne Occidentale, Brest, France; 19 Médecine Interne, Centre Hospitalier Universitaire Nantes, Nantes, France

**Keywords:** Haematological diseases, Epidemiology

## Abstract

Little is known about patients with sickle cell disease (SCD) who require intensive care unit (ICU) admission. The goals of this study were to assess outcomes in patients admitted to the ICU for acute complications of SCD and to identify factors associated with adverse outcomes. This multicenter retrospective study included consecutive adults with SCD admitted to one of 17 participating ICUs. An adverse outcome was defined as death or a need for life-sustaining therapies (non-invasive or invasive ventilation, vasoactive drugs, renal replacement therapy, and/or extracorporeal membrane oxygenation). Factors associated with adverse outcomes were identified by mixed multivariable logistic regression. We included 488 patients admitted in 2015–2017. The main reasons for ICU admission were acute chest syndrome (47.5%) and severely painful vaso-occlusive event (21.3%). Sixteen (3.3%) patients died in the ICU, mainly of multi-organ failure following a painful vaso-occlusive event or sepsis. An adverse outcome occurred in 81 (16.6%; 95% confidence interval [95% CI], 13.3%–19.9%) patients. Independent factors associated with adverse outcomes were low mean arterial blood pressure (adjusted odds ratio [aOR], 0.98; 95% CI 0.95–0.99; *p* = 0.027), faster respiratory rate (aOR, 1.09; 95% CI 1.05–1.14; *p* < 0.0001), higher haemoglobin level (aOR, 1.22; 95% CI 1.01–1.48; *p* = 0.038), impaired creatinine clearance at ICU admission (aOR, 0.98; 95% CI 0.97–0.98; *p* < 0.0001), and red blood cell exchange before ICU admission (aOR, 5.16; 95% CI 1.16–22.94; *p* = 0.031). Patients with SCD have a substantial risk of adverse outcomes if they require ICU admission. Early ICU admission should be encouraged in patients who develop abnormal physiological parameters.

## Introduction

Sickle cell disease (SCD) is a genetic disorder responsible for the presence of an abnormal type of haemoglobin, haemoglobin S (Hb S). SCD occurs chiefly in individuals of African or Mediterranean origin. In some circumstances, Hb S polymerises within the red blood cells (RBCs), which obstruct the small blood vessels, causing vaso-occlusive events (VOEs) characterised by ischaemia and severe pain. Over time, recurrent infarctions cause chronic organ failure. In the lung, this process is known as acute chest syndrome (ACS). SCD affects about 300,000 newborns each year worldwide^1^. The prevalence is increasing in Europe^2^ due to the migration of individuals from Africa and the Caribbean, as well as to increases in life expectancy as medical care improves. Thus, SCD is the most common genetic disorder in France^3^. SCD remains a life-threatening disease that shortens the life expectancy of patients by about 25 years compared to the general population^1^. The main causes of death are ACS, infection, stroke, and end-stage organ failure^4–6^.

Patients with SCD may require admission to the intensive care unit (ICU) in case of severe VOE^7^. Few studies of patients with SCD admitted to the ICU are available. The main reason for ICU admission is ACS, and ICU mortality has ranged from 7 to 19.6%^8,9^. Risk factors for mortality were older age, higher number of prior hospitalisations, longer ICU length of stay (LOS), use of mechanical ventilation and/or vasoactive drugs, a high haemoglobin level, a fast respiratory rate, and acute kidney injury (AKI) at ICU admission^1,5^. Blood transfusion in the ICU was a risk factor for death in one study^7^. However, most studies of risk factors were done in single centres, expert centres, or centres in low-income countries with a high prevalence of SCD and limited medical resources. Data collected by European teams diverge in terms of mortality rates and risk factors. Those divergences may be related to variations in admission policies despite national guidelines^3^.

We conducted a large multicenter retrospective study to describe the management and outcomes of patients with SCD admitted to ICUs in continental France. We also sought to identify factors associated with adverse outcomes.

## Patients and methods

### Study design and population

We conducted a multicenter retrospective study in 17 ICUs of French university hospitals. Consecutive adults with SCD admitted to the participating ICUs between 1 January 2015 and 31 December 2017 were included. Patients were followed up for 1 year after ICU discharge. Minors and patients with sickle cell trait (heterozygous for the causative gene) were not included.

Patients were identified by searching the hospital databases for code D57 in the International Classification of Diseases-10th revision then selecting those who required ICU admission. In patients with several admissions during the study period only the first admission was included.

### Data collection

For each patient, we manually collected the following from the medical records: demographic data, history of VOE (including the Hebbel score^10^) and of other acute complications, chronic complications, number of hospitalisations in the past year, history of blood transfusion, laboratory test results, and chronic treatments. We also recorded the following information about the hospital stay before ICU admission: reason for admission, vital parameters at admission, initial laboratory test results, and treatment. We collected the reason for ICU admission; acute organ failures defined as need for specific organ-supporting interventions; vital signs and laboratory test results at admission; and treatments including analgesics, blood support, antibiotics, and life-supporting interventions such as oxygen therapy, non-invasive ventilation (NIV), invasive mechanical ventilation (MV), renal replacement therapy (RRT), vasoactive drugs, and extracorporeal membrane oxygenation (ECMO). AKI was defined as a serum creatinine increase to at least 1.5-fold the baseline value. If the baseline value was unknown, it was estimated in patients without chronic kidney failure by calculating the serum creatinine level using the CKD-EPI (Chronic Kidney Disease EPIdemiology) formula without adjustment on ethnicity, for an estimated glomerular filtration rate (eGFR) of 75 mL/min/73 m^2^, according to international guidelines issued by the Kidney Disease Improving global Outcome Group (KDIGO)^11^. Hospital LOS, ICU LOS, and vital status at ICU discharge and hospital discharge were recorded. Finally, we collected the admissions and deaths that occurred during the 1-year follow-up.

All data were collected by the same investigator (MA), who was trained as a chart abstractor^12^. Regular meetings were performed between the chart abstractor and the study coordinators (NB, JBL). Disagreements were resolved after discussion among these three individuals.

### Outcomes

The primary outcome was a composite of adverse outcomes consisting of death in the ICU and/or the use of life-sustaining interventions (NIV, MV, RRT, vasopressors, and/or ECMO^13^. The secondary outcomes were the need for life-sustaining interventions, ICU mortality, hospital mortality, 1-year mortality, and 1-year re-admission rate.

### Statistical analysis

Quantitative data are described as mean ± standard deviation or median and interquartile range and qualitative data as numbers and percentages. Univariate analyses were conducted to identify variables potentially associated with the primary outcome. Variables with clinical relevance were chosen a priori and entered in a multivariable logistic regression model. They were baseline characteristics (age, sex, genotype, chronic kidney disease and number of hospitalisations in the past year), data on the hospital stay before ICU admission (reason for initial hospital admission and blood transfusion, RBC exchange, and length of stay [LOS] before ICU admission) and clinical and laboratory variables at ICU admission (mean blood pressure, respiratory rate, haemoglobin level, creatinine clearance, AKI, and total bilirubin level). They were entered by stepwise backward selection based on the Akaike information criterion^14^. The model was adjusted on centre as a random effect. We did not enter acute disease severity scores such as the SAPSII or SOFA score as they included data from the first 24 h in the ICU or combined different data that can be difficult to interpret at the bedside.

The statistical analyses were performed using SAS software version 9.4 (SAS Institute, Cary, NC, USA), and forest plots were generated using the R programme version 3.6 (R Foundation for Statistical Computing, Vienna, Austria; https://www.R-project.org/). No imputation strategy was used for missing data. Values of *p* < 0.05 were considered statistically significant.

### Ethics approval

The Ethics Committee of the French Intensive Care Society approved the study (CE SRLF #18-16). Informed consent was waived according to French law on retrospective studies of anonymised data (articles L.1121-1 paragraph 1 and R1121-2, Public Health Code). All methods were carried out in accordance with relevant guidelines and regulations.

### Consent to participate

Informed consent was waived according to French law on retrospective studies of anonymised data.

## Results

### Population

**Figure 1:**
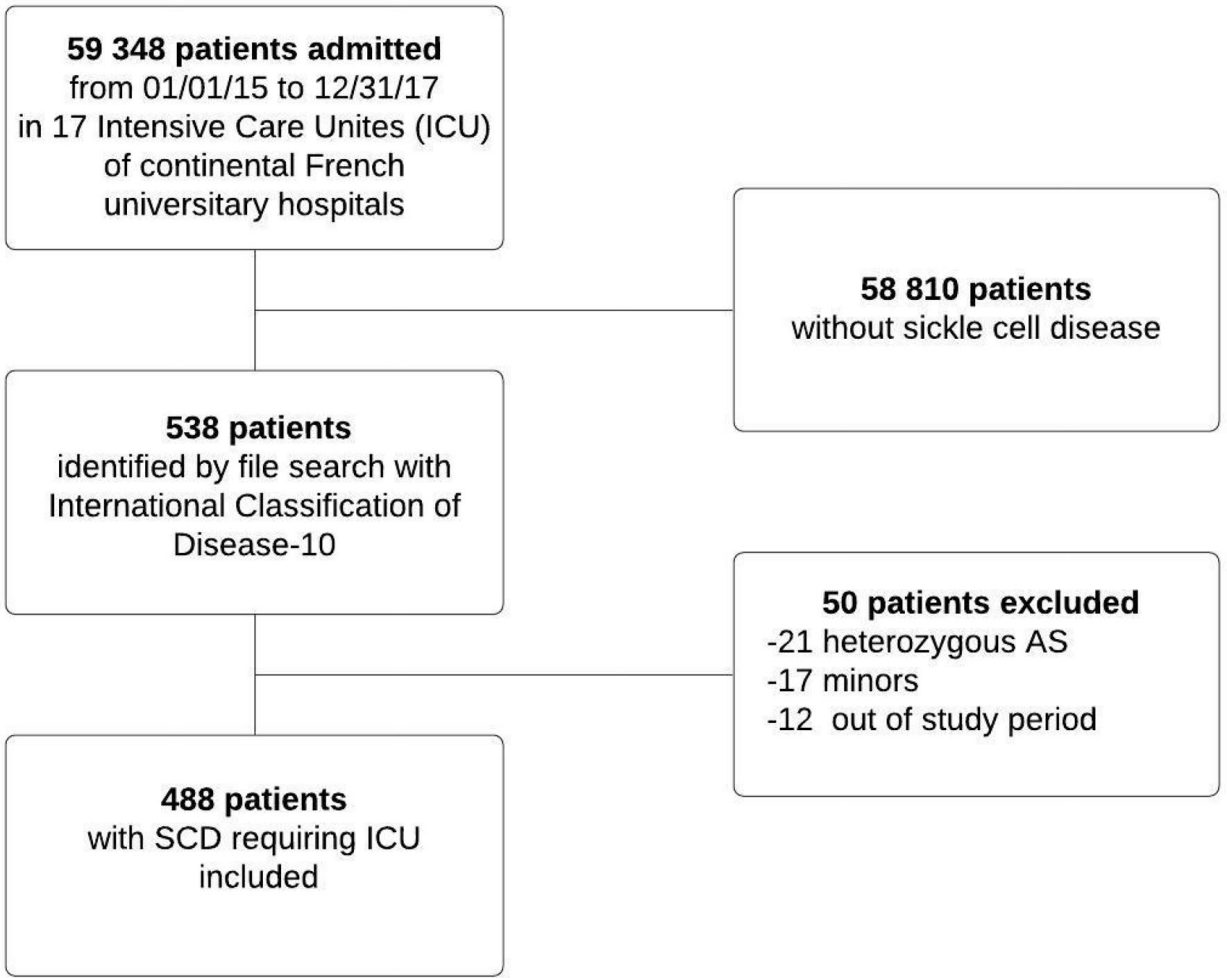
Patient flowchart.

From 1 January 2015 to 31 December 2017, 488 patients were included in the study (Fig. 1). Table 1 reports their baseline characteristics.

**Table 1:** Patient characteristics before hospital admission (stable status).

Characteristics	MD	All N = 488	AO N = 81	No AO N = 407	*p* value
Age (years), mean ± SD	0	31 ± 10.8	33.4 ± 12.5	30.6 ± 10.4	**0.04**
Females, n (%)	0	242 (49.5)	35 (43.2)	207 (50.9)	0.21
BMI (kg/m^2^), mean ± SD	112	22.5 ± 4.2	23.7 ± 5.2	22.2 ± 3.9	**0.02**
**Genotype, n (%)**	4				0.15
SS		418 (86.4)	65 (81.3)	353 (87.4)	
SC		27 (5.6)	7 (8.8)	20 (5)	
Sß^+^		22 (4.5)	6 (7.5)	16 (4)	
Sß^0^		12 (2.5)	0 (0)	12 (3)	
Other		5 (1)	2 (2.5)	3 (0.7)	
**Complications of SCD**, n (%)	3				
Acute chest syndrome		293 (60)	39 (48.2)	254 (62.6)	**0.02**
Avascular osteonecrosis		139 (28.5)	20 (24.7)	119 (29.3)	0.4
Chronic kidney failure		37 (7.6)	12 (14.8)	25 (1.2)	**0.009**
Pulmonary hypertension		25 (5.1)	8 (9.9)	17 (4.2)	**0.04**
Stroke		17 (3.5)	1 (1.2)	16 (3.9)	0.252
**Others complications of SCD**, n (%)	3				
Allo-immunisation		79 (16.2)	18 (22.2)	61 (15)	0.11
Delayed haemolytic transfusion reaction		39 (8)	9 (11.1)	30 (7.4)	0.27
Hebbel score, mean ± SD		2 ± 2	2 ± 2	3 ± 2	**0.01**
**Medical history**, **n (%)**	1				
Chronic arterial hypertension		30 (6.1)	12 (14.8)	18 (4.4)	** < 0.001**
Immunodepression		12 (2.5)	4 (4.9)	8 (2)	0.13
Diabetes mellitus		6 (1.2)	2 (2.5)	4 (1)	0.29
**Laboratory tests**, **median [IQR]**					
Haemoglobin (g/dL)	119	8.5 [7.8–9.5]	9 [7.7–10.0]	8.5 [7.8–9.5]	0.11
Creatinine (µmol/L)	351	56 [45–68]	68 [56–100]	54 [44–66]	0.14
ASAT (xN)	392	1 [1–1.3]	1 [1–1.3]	1 [1–1.4]	0.45
ALAT (xN)	393	1 [1–1]	1 [1–1]	1 [1–1]	0.36
Total bilirubin (µmol/L)	400	33 [21–53]	27 [24–40]	33 [20–61]	0.19
Indirect bilirubin (µmol/L)	342	17 [8–33]	17 [2, 3]	18 [8–35]	0.35
Direct bilirubin (µmol/L)	351	9 [7–14]	10 [9–11]	9 [7–14]	0.87
LDH (IU/L)	389	1.6 [1.4–2.1]	1.6 [1.2–2]	1.6 [1.4–2.2]	0.64
Haemoglobin S (%)	421	80 [61 -87]	74 [56–76]	80 [63–87]	0.49
**Chronic medication**, **n (%)**	2				
Folic acid		384 (79)	54 (66.7)	330 (81.5)	**0.003**
Hydroxyurea		240 (49.5)	36 (44.4)	204 (50.4)	0.33
ACE inhibitor		46 (9.7)	14 (17.3)	32 (7.9)	**0.01**
EPO		16 (3.3)	7 (8.6)	9 (2.2)	**0.006**
Penicillin		39 (8)	6 (7.4)	33 (8.1)	0.82
Iron chelation therapy		31 (6.4)	7 (8.6)	24 (5.9)	0.36
Transfusion therapy		31 (6.4)	8 (9.9)	23 (5.7)	0.16
**Medical history in the past year**	6				
All-cause hospitalisations, mean ± SD		1 ± 1.6	0.6 ± 1.1	1.1 ± 1.7	**0.03**
VOE-related hospitalisations, mean ± SD		0.9 ± 1.5	0.4 ± 0.9	0.9 ± 1.6	**0.03**
Blood transfusion^a^, n (%)		67 (13.9)	12 (14.9)	55 (13.7)	0.91

Significant values are in [bold].

*MD* missing data, *AO* adverse outcome defined as death in the ICU or use of life-sustaining treatments, *BMI* body mass index, *ACE* angiotensin-converting enzyme, *EPO* erythropoietin, *VOE* vaso-occlusive event, *SD* standard deviation, *IQR* interquartile range.

^a^Except prophylactic red blood cell exchange.

Table 2 reports the main clinical and laboratory test data of the patients, as well as the treatments, during the pre-ICU hospital stay. Table 3 shows data at ICU admission then in the ICU. Just before ICU admission, patients were either in the same hospital (emergency department, n = 196, 40.2%; medical ward, n = 238, 48.7%; surgical or obstetrics ward, n = 27, 5.5%; or another type of ward, n = 5, 1%) or in an ICU in another hospital (n = 22; 4.6%). The main reasons for initial hospital admission were VOE (n = 333; 82.8%) and ACS (n = 61; 12.5%). The median LOS prior to ICU transfer was 1 [0–3] day.

**Table 2:** Characteristics at hospital admission and process of care before ICU admission.

Characteristics Median [IQR] or n (%)	MD	All N = 488	AO N = 81	No AO N = 407	*p* value
Age	0	29 [23;37]	31 [22;40]	28 [23;36]	**0.0381**
Males	0	246 (50.41%)	46 (56.79%)	200 (49.14%)	0.21
**Admission diagnoses**	1				0.33
SCD-related acute VOE		403 (82.8)	64 (79)	339 (83.5)	
Non-SCD-related reason		76 (15.6)	19 (23.5)	57 (14)	
Scheduled hospitalisation		25 (5.1)	3 (3.7)	22 (5.4)	
**Vital parameters at hospital admission**					
MBP (mmHg)	180	90 [80–101]	90 [77–101]	90 [80–100]	0.92
Heart rate (beats/min)	200	89 [76–106]	100 [70–110]	89 [76–105]	0.31
Respiratory rate (breaths/min)	341	20 [16–24]	21 [15–28]	20 [16–24]	0.35
Oxygen saturation (%)	194	98 [95–100]	98 [95–100]	98 [95–100]	0.34
Visual pain scale	170	8 [7–10]	8 [7–10]	8 [7–10]	0.79
GCS score	345	15 [15–15]	15 [15–15]	15 [15–15]	0.05
Temperature (°C)	219	37.4 [36.6–37.7]	37.2 [36.7–38.3]	37.5 [36.6–37.6]	**0.02**
**Laboratory tests at hospital admission**					
Haemoglobin (g/dL)	74	8.6 [7.4–9.6]	8.5 [7–9.8]	8.6 [7.5–9.6]	0.38
Leucocytes (G/L)	111	14.9 [11.9–19]	15 [12.3–21.1]	14.8[11.7– 18.7]	0.22
Platelets (G/L)	134	320 [215–405]	287 [194–383]	328 [223–406]	0.14
Creatinine (µmol/L)	142	59 [48–74]	69 [55–86]	58 [47–72]	**0.003**
ASAT (xN)	167	1.2 [1, 2]	1.4 [1–2.1]	1.2 [1, 2]	0.64
ALAT (xN)	163	1 [1–1.5]	1 [1–1.7]	1 [1–1.5]	0.26
Total bilirubin (µmol/L)	151	44 [27–76]	47.5 [30–101]	44 [26–73]	0.11
Direct bilirubin (µmol/L)	237	11 [8–17]	12 [8–39]	11 [8–15]	**0.01**
Indirect bilirubin (µmol/L)	226	30 [16–54]	31 [18–63]	30 [16–53]	0.47
LDH (xN)	394	2.3 [1.7–3.1]	2.2 [1.8–3.4]	2.3 [1.7–3.1]	0.27
PT (%)		74 [64.5–86]	72 [60–83]	74 [63–86]	0.21
**Treatments before ICU admission**					
Opioids	14	376 (79.3)	57 (72.2)	319 (80.8)	0.09
Ketamine	15	42 (8.9)	3 (3.8)	14 (4.3)	0.09
NSAIDs	16	23 (4.9)	6 (7.6)	17 (4.3)	0.22
Blood transfusion	13	64 (13.5)	16 (20)	48 (12.2)	0.06
Number of RBC packs	12	2 [2, 3]	3.5 [2–4]	2 [2, 2]	** < 0.001**
Exchange transfusion	13	19 (4)	5 (6.3)	14 (3.5)	0.26
Number of RBC packs	12	2 [2, 3]	2 [2–2]	2 [2, 3]	0.75
Bloodletting	11	8 (1.7)	1 (1.3)	7 (1.8)	**0.04**
Steroids		9 (1.9)	4 (5)	5 (1.3)	**0.001**
Antibiotics		227 (47.6)	52 (64.2)	175 (44.2)	
**LOS before ICU admission**	0	1 [0–3]	2 [1–3]	1 [0–3]	**0.04**

Significant values are in [bold].

The *p* values were obtained by univariate logistic regression and reflect associations with the occurrence of adverse outcomes.

*IQR* interquartile range, *MD* missing data, *AO* adverse outcome defined as death or use of life-sustaining treatments, *SCD* sickle cell disease, *MBP* mean arterial blood pressure, *GCS* Glasgow Coma Scale, *ASAT* aspartate transaminase, *ALAT* alanine transaminase, *LDH* lactate dehydrogenase, *PT* prothrombin time, *ICU* intensive care unit, *NSAIDs* nonsteroidal anti-inflammatory drugs, *RBC* red blood cells, *LOS* length of stay, *ACS* acute chest syndrome, *VOE* vaso-occlusive event, *NIV* non-invasive ventilation, *MV* endotracheal mechanical ventilation, *RRT* renal replacement therapy, *ECMO* extracorporeal membrane oxygenation, *SAPS II* Simplified Acute Physiology Score II.

**Table 3:** Characteristics at ICU admission and process of care during the ICU stay.

Characteristics Median [IQR] or n (%)	MD	All N = 488	AO N = 81	No AO N = 407	*p* value
**ICU admission diagnoses**	0				
ACS		232 (47.5)	40 (49.4)	192 (47.2)	0.7
Severe VOE		104 (21.3)	4 (4.9)	100 (24.6)	** < 0.0001**
Sepsis ^a^		42 (8.6)	17 (21.3)	25 (6.1)	** < 0.0001**
Pulmonary embolism		18 (3.7)	5 (6.3)	13 (3.2)	0.19
Drug /morphine overdose		12 (2.5)	3 (3.7)	9 (2.2)	0.42
Other VOE^b^		83 (17)	13 (16)	70 (17.3)	0.8
Other diagnoses^c^		48 (9.8)	17 (21)	31 (7.6)	**0.0002**
**Acute organ failure**	3				
Acute respiratory distress		116 (23.9)	31 (38.8)	85 (21)	**0.0008**
Shock		14 (2.9)	6 (7.5)	8 (2)	**0.02**
Coma		15 (3.1)	8 (10)	7 (1.7)	** < 0.001**
Acute kidney injury		3 (0.6)	1 (1.2)	2 (0.5)	0.42
Multi-organ failure		7 (1.4)	7 (8.8)	0 (0)	** < 0.00001**
**Vital parameters at ICU admission**					
MBP (mmHg)	29	90 [80–100]	87 [76–100]	90 [80–99]	0.3
Heart rate (beats/min)	31	100 [84–115]	110 [96–125]	97 [82–112]	** < 0.0001**
Respiratory rate (breaths/min)	117	24 [18–30]	28 [23–32]	23 [17–28]	** < 0.0001**
Oxygen saturation (%)	41	98 [96–100]	98 [96–100]	99 [96–100]	0.13
Visual pain scale	226	7 [4–8]	7 [3–8]	7 [4–8]	0.48
GCS score	42	15 [15–15]	15 [14, 15]	15 [15–15]	** < 0.001**
Temperature (°C)	73	37.4[36.9– 38.2]	37.7 [37–38.6]	37.3[36.9– 38.1]	0.33
**Laboratory tests at ICU admission**					
Haemoglobin (g/dL)	10	8 [6.7–9.1]	8.1 [6.2–9.4]	8 [6.7–9.1]	0.68
Leucocytes (G/L)	17	15.1 [11–20]	17 [12.7–24]	15 [11.2–20]	** < 0.0001**
Platelets (G/L)	12	264 [170–360]	192 [123–303]	276 [183–374]	**0.0002**
Creatinine (µmol/L)	10	54 [41–72]	75 [53–173]	52 [40–66]	** < 0.0001**
PT (%)	45	68 [57–78]	61 [36–71]	69 [60–78]	** < 0.0001**
Arterial lactate (mmol/L)	44	0.9 [0.6–1.4]	1.8 [1–3.5]	0.8 [0.6–1.2]	** < 0.0001**
Haemoglobin S (%)	47	77 [59–85]	68.1 [41.5–75]	78.9 [60.9 -85.3]	** < 0.0001**
Haemolytic parameters:	118	1.3 [1–2.3]	2.1 [1.2–5]	1.2 [1, 2]	0.99
ASAT (xN)	125	1 [1–1.7]	1 [1, 2]	1 [1–1.6]	0.42
ALAT (xN)	197	41 [24–77]	58 [27–103]	39 [23–71]	0.16
Total bilirubin (µmol/L)	221	14 [8–28]	22.5 [11–53]	13 [823]	**0.009**
Direct bilirubin (µmol/L)	336	2.4 [1.7–4.6]	3.6 [2–10.3]	2.3 [1.6–3.7]	**0.002**
LDH (xN)					
**Treatment in the ICU**					
Opioids	0	391 (80.1)	60 (74.1)	331 (81.3)	0.14
Ketamine	0	115 (23.6)	9 (11.1)	106 (26)	**0.005**
NSAIDs	0	26 (5.3)	1 (1.2)	25 (6.1)	0.11
Blood transfusion	0	127 (26)	33 (40.7)	94 (23.1)	**0.001**
Number of RBC packs	0	0 [0–2]	2 [0–2]	0 [0–2]	0.518
Exchange transfusion	0	114 (23.3)	21 (25.9)	93 (22.8)	0.23
Number of RBC packs	0	4 [2–5]	0 [0–4]	2 [2–4]	**0.0006**
Bloodletting		12 (2.5)	0 (0)	12 (3)	**0.003**
Steroids	0	15 (3.1)	8 (9.9)	7 (1.7)	
Antibiotics		361 (73.8)	71 (87.7)	289 (71)	
**Life-supporting treatments**	0				
NIV		29 (5.9)	29 (35.8)	0 (0)	
MV		46 (9.4)	46 (56.8)	0 (0)	
Duration (days)		5 [2–10]	5 [2–10]	0 (0)	
Vasoactive drugs		32 (6.6)	32 (39.5)	0 (0)	
RRT		18 (3.7)	18 (22.2)	0 (0)	
Veno-venous ECMO		5 (1)	5 (6.2)	0 (0)	
Arterio-venous ECMO		3 (0.6)	29 (35.8)		
**SAPS II**	88	16 [10–25]	34 [24–41]	15 [10–22]	** < 0.0001**

Significant values are in [bold].

The *p* values were obtained by univariate logistic regression and reflect associations with the occurrence of adverse outcomes.

*IQR* interquartile range, *MD* missing data, *AO* adverse outcome defined as death or use of life-sustaining treatments, *SCD* sickle cell disease, *MBP* mean arterial blood pressure, *GCS* Glasgow Coma Scale, *ASAT* aspartate transaminase, *ALAT* alanine transaminase, *LDH* lactate dehydrogenase, *PT* prothrombin time, *ICU* intensive care unit, *NSAIDs* nonsteroidal anti-inflammatory drugs, *RBC* red blood cells, *LOS* length of stay, *ACS* acute chest syndrome, *VO* vaso-occlusive, *VOE* vaso-occlusive event, *NIV* non-invasive ventilation, *MV* endotracheal mechanical ventilation, *RRT* renal replacement therapy, *ECMO* extracorporeal membrane oxygenation, *SAPS II* Simplified Acute Physiology Score II.

^a^Sepsis was suspected when mentioned in medical records and confirmed when the patient had an infection accompanied with a life-threatening organ dysfunction.

^b^Including delayed haemolytic transfusion reaction, stroke, acute anaemia, priapism, splenic sequestration, VOE without severe pain, and VOE after surgery or childbirth.

^c^Including cardiac arrest, pulmonary oedema, acute coronary syndrome, exacerbation of chronic respiratory failure, seizure and status epilepticus, acute kidney failure, metabolic disorders, pancreatitis, hepatitis, gastro-intestinal bleeding, gastro-intestinal occlusion, mesenteric ischaemia, haematologic disorders, anaphylaxis, and peri-partum complications.

The main reasons for ICU admission were ACS (n = 232; 47.5%), VOE with severe pain (n = 104; 21.3%), sepsis (n = 42; 8.6%), and pulmonary embolism (n = 18; 3.7%). The sites of infection in patients with sepsis were the lungs (25%), abdomen (20.5%), central nervous system (11.4%), bloodstream (9.1%), urinary tract (6.8%), upper respiratory tract or ears (6.8%), and bones and joints (6.8%). Blood support was required by 240 (49.3%) patients (blood transfusion, n = 127, 26%; or RBC exchange, n = 114, 23.3%). Antibiotics were administered to 361 (73.8%) patients and consisted chiefly of cephalosporins (n = 259; 53.1%), macrolides (n = 231; 44.9%), and penicillins (n = 79; 16.2%). The bacteria identified are listed in the supplementary material file 1 (ESM 1).

### Primary outcome

Of the 488 patients, 81 (16.6%; 95% confidence interval [95% CI], 13.3–19.9%) met our composite adverse-outcome criterion. Among them, all required life-supporting treatments, and 16 (16/488, 3.3%) died. The reasons for requiring MV were ACS and/or acute respiratory distress syndrome (41.3%), neurologic failure (17.4%), multi-organ failure (13%) shock (8.7%), cardiac arrest (8.7%), scheduled surgery or investigation (8.7%), and other (2.2%).

### Factors associated with the composite adverse-outcome criterion

Tables 1, 2 and 3 report the factors associated with adverse outcomes by univariate analysis. Patients who experienced adverse outcomes were significantly older and more often had comorbidities (hypertension, chronic kidney disease, and/or pulmonary hypertension) but less often had a previous history of ACS compared to patients without adverse outcomes.

**Figure 2:**
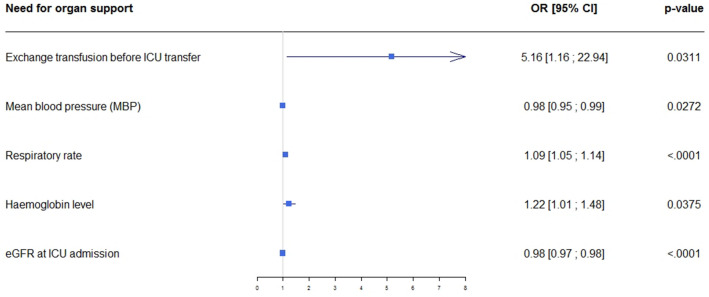
Forest plot of factors associated with adverse outcomes by multivariable analysis.

By multivariable analysis adjusted on centre, factors independently associated with a need for life-supporting treatments were RBC exchange before ICU admission, lower mean blood pressure (MBP), higher respiratory rate, higher haemoglobin level, and lower creatinine clearance at ICU admission (Fig. 2). LOS before ICU transfer was not independently associated with adverse outcomes. Distribution of patients with need for organ support according to centre is displayed on ESM 2.

### Patients who died and 1-year outcomes in survivors

Median ICU LOS was 5 [3–8] days, and median hospital LOS was 12 [9–16] days (Table 4). Of the 488 patients, 16 (3.3%) died in the ICU and 18 (3.7%) in the hospital. Their profiles are detailed in the supplementary material file 2 (ESM 3). Two profiles emerged: half the patients who died were older than the median age of the cohort and had chronic SCD complications such as chronic kidney failure or another significant SCD-related condition, whereas the other half were younger patients with no history of SCD complications and a Hebbel score of 0. Of note, a third of the patients who died had a genotype other than homozygous SS (ESM 2).

**Table 4:** Outcome at discharge and during the one-year follow up.

Characteristics Median [IQR] or n (%)	MD	All N = 488	AO	No AO	*p* value
ICU LOS (days)	0	5 (3–8)	6 [3–12]	5 [3–7]	**0.0001**
Hospital LOS (days)	14	12 [9–16]	14 [11–13]	11 [8–16]	
Re-admissions in the ICU during the following year	58	129 (29.9)	18 (27.3)	111 (30.5)	
Time to re-admission (days)	5	131 [31–244]	175 [86–279]	126 [14–239]	
ICU mortality	0	16 (3.3)	16 (19.8)	0 (0)	
Hospital mortality	0	18 (3.7)	17 (21)	1 (0.2)	
One-year mortality	58	27 (6.3)	20 (29)	7 (2)	

Significant values are in [bold].

The *p* values were obtained by univariate logistic regression and reflect associations with the occurrence of adverse outcomes. As “adverse outcome” was a composite criterion including death, no statistical tests were performed for re-admissions, ICU mortality, hospital mortality, or one-year mortality.

*IQR* interquartile range, *MD* missing data, *AO* adverse outcome defined as death or use of life-sustaining treatments, *SCD* sickle cell disease, *MBP* mean arterial blood pressure, *GCS* Glasgow Coma Scale, *ASAT* aspartate transaminase, *ALAT* alanine transaminase, *LDH* lactate dehydrogenase, *PT* prothrombin time, *ICU* intensive care unit, *NSAIDs* nonsteroidal anti-inflammatory drugs, *RBC* red blood cells, *LOS* length of stay, *ACS* acute chest syndrome, *VOE* vaso-occlusive event, *NIV* non-invasive ventilation, *MV* endotracheal mechanical ventilation, *RRT* renal replacement therapy, *ECMO* extracorporeal membrane oxygenation, *SAPS II* Simplified Acute Physiology Score II.

During the 1-year follow-up, 129 (30%) of the 454 survivors required ICU admission. The mean number of hospital stays per patient was 1.3, ranging from 1 to 7 during the study period. The 9 patients who died during follow up had all been readmitted, and all died in the ICU (global 1-year mortality, 27/488, 6%).

## Discussion

The aim of our large multicenter study was to assess the acute illness severity of patients with SCD admitted to the ICU and to identify factors associated with adverse outcomes defined as death in the ICU and/or use of life-supporting treatments. ACS accounted for nearly half the ICU admissions. The ICU mortality rate was 3.3%. About one out of six patients experienced adverse outcomes. Factors independently associated with adverse outcomes were RBC exchange prior to ICU admission, lower mean arterial blood pressure, faster respiratory rate, higher haemoglobin level, and lower creatinine clearance at ICU admission. After ICU discharge, almost a third of the patients required ICU admission during the following year.

In other studies, ACS was the reason for ICU admission in 30% to 70% of patients^7,8,13,15,16^. This wide range can be explained by differences in types of ICUs, study populations, and varying proportions of medical and surgical admissions. However, ACS is consistently reported as the main reason for ICU admission, in keeping with our findings. Other studies found mortality rates of 12–22%^7,8,15,17^, compared to 3.3% in our study. The higher rates were found in older studies done in countries where the prevalence of SCD is high and healthcare resources limited. In a retrospective study done in France in patients managed between 2004 and 2010^13^, mortality was 7% (9/138). Patients with SCD may be unevenly distributed across France, with tertiary centres and referral centres admitting those patients with the greatest disease severity. Our study shows that the ICU mortality rate of patients with SCD is low in French tertiary centres. As previously reported^13^, we identified two profiles among the patients who died. Being young and relatively free of past SCD-related events did not constitute protection against fatal complications. A third of the patients who died were not homozygous for SCD, and genotype did not independently predict adverse outcomes as defined for our study. Therefore, genotype should not be used to assess the risk of complications. Finally, it is noteworthy that 30% of the survivors were re-admitted to the ICU within the following year. Patients with SCD have a high rate of emergency-department and hospital admissions and re-admissions^18^. The 30-day readmission rate is used in children as an indicator of quality of care^19^. A study has shown that intensive management in a referral clinic lowered admissions of patients with more than 12 emergency-department or hospital admissions per year^20^.

As previously reported, a high respiratory rate and impaired creatinine clearance at ICU admission were associated with adverse outcomes^16,19,20^. A high haemoglobin level was also associated with adverse outcomes, whereas previous studies showed that a low haemoglobin level predicted a poor prognosis^13,16,21^. This discrepancy may be related to the administration of blood support before ICU admission in some patients in our study. However, this subgroup accounted for only 17.5% of all patients, and the haemoglobin level at ICU admission was not significantly different between groups with and without adverse outcomes. Two, heretofore unreported factors associated with adverse outcomes were lower MBP and RBC exchange before ICU admission. MBP can decrease due to sepsis or to severe VOE with acute pulmonary hypertension, which can progress to cor pulmonale and multi-organ failure^22,23^. Finally, among factors occurring before ICU admission, LOS was not a predictor, whereas RBC exchange was independently associated with adverse outcomes. RBC exchange is restricted to a limited number of situations that complicate the most severe VOEs^24,25^. The risk of sudden worsening has been taken to warrant broad ICU referral of patients with SCD-related VOEs^18^. Our data suggest that consideration for prompt ICU admission may be warranted in patients admitted for VOEs and requiring RBC exchange. This suggestion may be incorporated in the next national guidelines^3^.

Our study has several strengths. First it is the first multicentre study conducted nationwide in France to assess adult patients with SCD admitted to the ICU. Our population is representative of patients managed in tertiary centres in continental France, as opposed to SCD-referral centres. Second, our cohort is large compared to other studies. Furthermore, we collected data on consecutive patients admitted to the ICU, regardless of the reason for admission, to obtain a comprehensive picture of the population of patients with SCD in the ICU. Finally, the 1-year follow-up after ICU discharge provided information on delayed morbidity and mortality.

The limitations of our study include the retrospective design, which led to missing data, especially for baseline characteristics (especially regarding history of pulmonary hypertension). Second, we did not include centres from overseas French departments, where the prevalence of SCD is higher. However, most patients with SCD in France live in the Paris area, and we included two referral centres, making our cohort representative of patients on French territory. Third, we did not include surgical ICUs and we therefore had few post-surgical patients. VOEs are common after surgery in patients with SCD^26^. We collected data at hospital admission and at ICU admission, but we did not compare the values at these two time points or determine whether the values changed within a few hours of ICU admission. However, 40% of patients were admitted to the ICU form the emergency department on the day of admission or on the next day. We did not assess echocardiography findings, although acute pulmonary hypertension is a severity factor in ACS^16^. However, echocardiography is not performed routinely in patients with ACS in all centres. We did not evaluate the frequency of high flow nasal oxygen (HFNO) therapy, which is increasingly used to treat SCD-related VOEs^27^. A trial comparing HFNO to standard oxygenation to prevent ACS during VOE is in progress (NCT03976180). We adjusted the estimated glomerular filtration rate on ethnicity, which may not be the best determination method^28,29^. Last, patients were included between 2015 and 2017, but the management of patients with SCD changes continuously. However new treatments are not easily available^30,31^ at the bedside and/or adopted by national guidelines.

## Conclusion

Our work shows that mortality in tertiary-centre ICUs is low in patients with SCD but that life-supporting treatments are often required despite the young age of the population. Furthermore, we found that some of the patients who died were young and had experienced few SCD-related events in the past. RBC exchange in the hospital before ICU admission was an independent risk factor for adverse outcomes, suggesting that patients who need RBC exchange should be considered for prompt admission to the ICU, where they can be monitored closely.

## Supplementary Information

Supplementary Information 1.

Supplementary Information 2.

Supplementary Information 3.

## Data Availability

The study data will be made available upon reasonable request to the corresponding author.

## References

[1] Ware RE, de Montalembert M, Tshilolo L, Abboud MR (2017). Sickle cell disease. Lancet Lond. Engl..

[2] Modell B, Darlison M, Birgens H, Cario H, Faustino P, Giordano PC (2007). Epidemiology of haemoglobin disorders in Europe: An overview. Scand. J. Clin. Lab. Invest..

[3] Habibi A, Arlet J-B, Stankovic K, Gellen-Dautremer J, Ribeil J-A, Bartolucci P (2015). Recommandations françaises de prise en charge de la drépanocytose de l’adulte : Actualisation 2015. Rev. Méd.. Int..

[4] Manci EA, Culberson DE, Yang Y-M, Gardner TM, Powell R, Haynes J (2003). Causes of death in sickle cell disease: An autopsy study. Br. J. Haematol..

[5] Perronne V, Roberts-Harewood M, Bachir D, Roudot-Thoraval F, Delord J-M, Thuret I (2002). Patterns of mortality in sickle cell disease in adults in France and England. Hematol. J..

[6] Platt OS, Brambilla DJ, Rosse WF, Milner PF, Castro O, Steinberg MH (1994). Mortality in sickle cell disease. Life expectancy and risk factors for early death. N. Engl. J. Med..

[7] Gardner K, Douiri A, Drasar E, Allman M, Mwirigi A, Awogbade M (2016). Survival in adults with sickle cell disease in a high-income setting. Blood.

[8] Tawfic QA, Kausalya R, Al-Sajee D, Burad J, Mohammed AK, Narayanan A (2012). Adult sickle cell disease: A five-year experience of intensive care management in a university hospital in Oman. Sultan Qaboos Univ. Med. J..

[9] Abd Rahman R, DeKoninck P, Murthi P, Wallace EM (2018). Treatment of preeclampsia with hydroxychloroquine: A review. J. Matern-Fetal Neonatal Med..

[10] Hebbel RP, Boogaerts MA, Eaton JW, Steinberg MH (1980). Erythrocyte adherence to endothelium in sickle-cell anemia. A possible determinant of disease severity. N. Engl. J. Med..

[11] Khwaja A (2012). KDIGO Clinical Practice Guidelines for Acute Kidney Injury. Nephron.

[12] Gilbert EH, Lowenstein SR, Koziol-McLain J, Barta DC, Steiner J (1996). Chart reviews in emergency medicine research: Where are the methods?. Ann. Emerg. Med..

[13] Cecchini J, Lionnet F, Djibré M, Parrot A, Stojanovic KS, Girot R (2014). Outcomes of adult patients with sickle cell disease admitted to the ICU: A case series*. Crit. Care Med..

[14] Akaike H, Parzen E, Tanabe K, Kitagawa G (1998). Information theory and an extension of the maximum likelihood principle. Sel Pap Hirotugu Akaike.

[15] Al Khawaja SA, Ateya ZM, Al Hammam RA (2017). Predictors of mortality in adults with Sickle cell disease admitted to intensive care unit in Bahrain. J. Crit. Care..

[16] Mekontso Dessap A, Fartoukh M, Machado RF (2017). Ten tips for managing critically ill patients with sickle cell disease. Intensive Care Med..

[17] Godeau B, Brun-Buisson C, Roudot-Thoraval F, Ruivard M, Durègne L, Lefort Y (1999). Évolution et facteurs pronostiques des adultes atteints d’un syndrome drépanocytaire majeur lors de leur admission en réanimation. Rev. Méd. Int..

[18] Brousseau DC, Owens PL, Mosso AL, Panepinto JA, Steiner CA (2010). Acute care utilization and rehospitalizations for sickle cell disease. JAMA.

[19] Frei-Jones MJ, Field JJ, DeBaun MR (2009). Risk factors for hospital readmission within 30 days: A new quality measure for children with sickle cell disease. Pediatr. Blood Cancer.

[20] Koch KL, Karafin MS, Simpson P, Field JJ (2015). Intensive management of high-utilizing adults with sickle cell disease lowers admissions. Am. J. Hematol..

[21] Simonson JL, Rosentsveyg JA, Schwartz NG, Agrawal A, Koenig S, Zaidi GZ (2020). Hemoglobin target and transfusion modality for adult patients with sickle cell disease acute chest syndrome. J. Intensive Care Med..

[22] Mekontso Dessap A, Leon R, Habibi A, Nzouakou R, Roudot-Thoraval F, Adnot S (2008). Pulmonary hypertension and Cor pulmonale during severe acute chest syndrome in sickle cell disease. Am. J. Respir. Crit. Care Med..

[23] Yawn BP, Buchanan GR, Afenyi-Annan AN, Ballas SK, Hassell KL, James AH (2014). Management of sickle cell disease: Summary of the 2014 evidence-based report by expert panel members. JAMA.

[24] Lionnet F, Arlet J-B, Bartolucci P, Habibi A, Ribeil J-A, Stankovic K (2009). Recommandations pratiques de prise en charge de la drépanocytose de l’adulte. Rev. Méd. Int..

[25] Haberkern CM, *et al*. Cholecystectomy in Sickle Cell Anemia Patients: Perioperative Outcome of 364 Cases From the National Preoperative Transfusion Study 11. 9057634

[26] Adjepong KO, Otegbeye F, Adjepong YA (2018). Perioperative management of sickle cell disease. Mediterr. J. Hematol. Infect. Dis..

[27] Ricard J-D, Roca O, Lemiale V, Corley A, Braunlich J, Jones P (2020). Use of nasal high flow oxygen during acute respiratory failure. Intensive Care Med..

[28] Arlet J-B, Ribeil J-A, Chatellier G, Eladari D, De Seigneux S, Souberbielle J-C (2012). Determination of the best method to estimate glomerular filtration rate from serum creatinine in adult patients with sickle cell disease: A prospective observational cohort study. BMC Nephrol..

[29] Zelnick LR, Leca N, Young B, Bansal N (2021). Association of the estimated glomerular filtration rate with vs without a coefficient for race with time to eligibility for kidney transplant. JAMA Netw. Open.

[30] Niihara Y, Miller ST, Kanter J, Lanzkron S, Smith WR, Hsu LL (2018). A phase 3 trial of l-glutamine in sickle cell disease. N. Engl. J. Med..

[31] Ataga KI, Kutlar A, Kanter J, Liles D, Cancado R, Friedrisch J (2017). Crizanlizumab for the prevention of pain crises in sickle cell disease. N. Engl. J. Med..

