# Supplementary material for: Nationwide retrospective study of critically ill adults with sickle cell disease in France
Source: Sci Rep. 2021 Nov 30;11:23132. doi: 10.1038/s41598-021-02437-2 (PMC8632921; doi:10.1038/s41598-021-02437-2)
Supplement: Supplementary file 1 — Supplementary Information 1. [file 41598_2021_2437_MOESM1_ESM.docx]

**ESM 1. Infective agents identified in samples taken during the ICU stay**

| Type of sample | Infective agent |
| --- | --- |
| Blood, n= 33 | |
|  | Methicillin-susceptible *Staphylococcus aureus* n=7  *Escherichia coli* n= 5  *Staphylococcus epidermidis* n= 4  *Pseudomonas aeruginosa* n= 3  *Streptococcus pneumoniae* n= 2  *Proteus mirabilis* n= 2  *Streptococcus pyogenes* n= 1  *Enterococcus faecalis* n= 1  *Enterococcus aerogenes* n= 1  *Klebsiella pneumoniae* n= 1  Other n=6 |
| Urine, n= 16 | |
|  | *Escherichia coli* n= 9  *Enterococcus faecalis* n= 3  *Klebsiella pneumoniae* n= 3  *Citrobacter koseri* n= 1 |
| Sputum, n= 40 | |
|  | Methicillin-susceptible *Staphylococcus aureus* n= 19  Methicillin-resistant *Staphylococcus aureus* n= 3  *Pseudomonas aeruginosa* n= 3  *Streptococcus pneumoniae* n= 2  *Streptococcus pyogenes* n= 2  *Klebsiella pneumoniae* n= 2  *Proteus mirabilis* n= 2  *Streptococcus agalactiae* n= 1  *Streptococcus constellatus* n= 1  *Haemophilus influenzae* n= 1  *Enterococcus aerogenes* n= 1  *Acinetobacter baumannii* n= 1  Other n=2 |
| Bronchoalveolar lavage fluid, n= 8 | |
|  | *Pseudomonas aeruginosa* n= 3  Methicillin-susceptible *Staphylococcus aureus* n= 2  *Escherichia coli* n= 2  *Streptococcus pneumoniae* n= 1  *Klebsiella pneumoniae* n= 1  *Bacteroides fragilis* n=1  Other n= 1 |
| Cerebrospinal fluid, n= 3 | |
|  | *Streptococcus pneumoniae* n= 2  *Escherichia coli* n= 1 |
| Other, n= 32 | |
|  | Methicillin-susceptible *Staphylococcus aureus* n= 5  *Plasmodium falciparum* n= 3  *Streptococcus pyogenes* n= 2  *Pseudomonas aeruginosa* n= 2  *Enterobacter cloacae* n= 2  *Legionella pneumophila* n= 2  Coagulase-negative staphylococcus n= 1  *Streptococcus viridans* n= 1  *Enterococcus faecium* n= 1  *Klebsiella pneumoniae* n= 1  *Clostridium paraputrificum* n= 1  *Toxoplasma gondii* n= 1  *Candida albicans* n= 1  Virus^a^ n=9 |

^a^Respiratory syncytial virus, n=2; Cytomegalovirus, n=2; Influenza B, n=1; coronavirus, n=1; parvovirus B19, n=2; rhinovirus, n=1
