# Supplementary figures and images for: Nationwide retrospective study of critically ill adults with sickle cell disease in France
Source: Sci Rep. 2021 Nov 30;11:23132. doi: 10.1038/s41598-021-02437-2 (PMC8632921; doi:10.1038/s41598-021-02437-2)

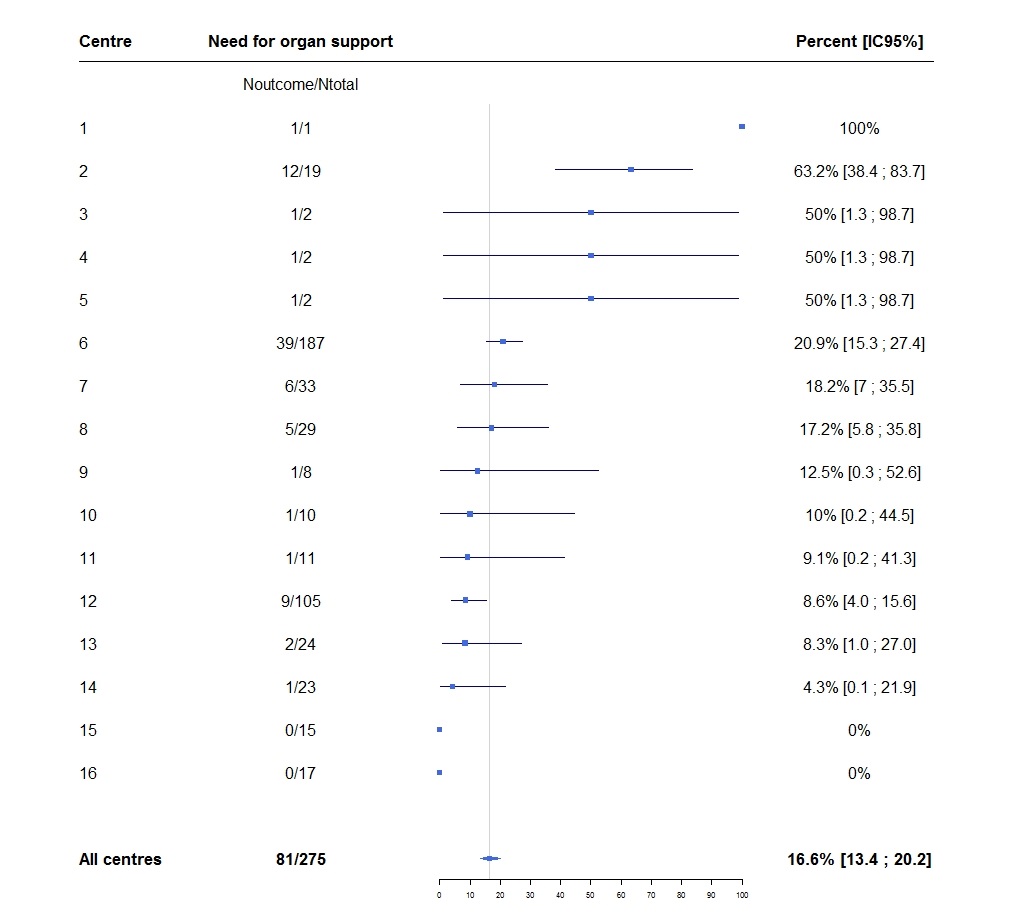

Supplement: Supplementary file 2 — Supplementary Information 2. [file 41598_2021_2437_MOESM2_ESM.jpeg]
