# Supplementary material for: Nationwide retrospective study of critically ill adults with sickle cell disease in France
Source: Sci Rep. 2021 Nov 30;11:23132. doi: 10.1038/s41598-021-02437-2 (PMC8632921; doi:10.1038/s41598-021-02437-2)
Supplement: Supplementary file 3 — Supplementary Information 3. [file 41598_2021_2437_MOESM3_ESM.docx]

ESM2. Characteristics of the 16 patients who died

| **Patient** | **1** | **2** | **3** | **4** | **5** | **6** | **7** | **8** | **9** | **10** | **11** | **12** | **13** | **14** | **15** | **16** |
| --- | --- | --- | --- | --- | --- | --- | --- | --- | --- | --- | --- | --- | --- | --- | --- | --- |
| Age (y) | 61 | 26 | 29 | 24 | 41 | 19 | 34 | 47 | 18 | 20 | 23 | 39 | 53 | 56 | 29 | 19 |
| Sex | F | M | F | F | M | F | M | M | F | M | M | F | M | M | M | M |
| Genotype | SS | SB+ | SS | SS | SS | SS | SS | SS | SB+ | SS | SC | SS | SC | SS | SC | SS |
| Conditions | CKD, PAH |  | ACS |  | CKD |  | ACS | ACS | ACS |  |  | ACS |  | ACS, CKD |  | ACS |
| Hebbel score | 0 | 0 | 4 | 0 | 0 | 3 | 3 | 3 | 2 | 0 | 0 | 4 | 0 | 6 | 0 | 2 |
| Admissions in past year, n | 3 | 0 | 1 | 0 | 0 | 1 | 1 | 0 | 0 | 0 | 0 | 0 | 0 | 0 | 0 | 0 |
| Baseline haemoglobin (g/dL) | 8.6 | 14.5 | 7.5 | MD | MD | MD | 8,2 | MD | MD | 11 | 8 | 9 | MD | 9 | 13 | 8 |
| Reason for hospital admission | VOC | Menin-gism | VOC | VOC | Surgery | ACS | Confu-sion | VOC | VOC  Pneu-monia | Pneu-monia | VOC | VOC | Hepa-titis | VOC | VOC | ACS |
| Reason for ICU admission | Other | Sepsis | drug poison-ing | Other | Haemor-ragic shock | ACS / sepsis | sepsis | DHTR | ACS  PE | sepsis | CHF | VOC | Acute liver failure | sepsis | ACS | ACS |
| Organ failure | 0 | coma | coma | MOF | MOF | 0 | 0 | MOF | ARD | 0 | ARD | MOF | MOF | Coma | MOF | ARD |
| RR (cpm) | 27 | MD | MD | 25 | 30 | 21 | 24 | 34 | 40 | MD | 30 | MD | MD | 30 | 24 | 30 |
| AKI | NO | NO | NO | YES | NO | NO | NO | YES | NO | NO | NO | NO | NO | YES | NO | NO |
| RBCx before ICU | NO | NO | NO | NO | NO | NO | NO | NO | NO | NO | NO | NO | YES | NO | NO | YES |
| ICU LOS, days | 1 | 0 | 5 | 0 | 2 | 1 | 0 | 2 | 2 | 0 | 0 | 3 | 1 | 2 | 2 | 10 |
| Hospital LOS, days | 8 | 9 | 5 | 1 | 53 | 1 | 5 | 3 | 3 | 1 | 5 | 8 | 3 | 12 | 12 | 13 |
| Cause of death | MOF | Sudden cardiac arrest | Sudden cardiac arrest | Sudden cardiac arrest | MOF | PE | Sudden cardiac arrest | MOF | Cor pul-monale | Septic shock | Haemorragic shock | MOF | MOF | Septic shock + MOF | MOF | MOF |

ACS; acute chest syndrome; AKF: acute kidney failure; ARD: acute respiratory distress; CHF: congestive heart failure; CKD: chronic kidney disease; DHTR: delayed haemolytic transfusion reaction; RBCx: red-blood-cell exchange transfusion; ICU: intensive care unit; LOS: length of stay; MD: missing data; MOF: multiorgan failure; PAH: pulmonary arterial hypertension; PE: pulmonary embolism; RR: respiratory rate; cpm: cycles per minute; VOC: vaso-occlusive crisis
